# Efficient production of pronuclear embryos in breeding and nonbreeding season for generating transgenic sheep overexpressing TLR4

**DOI:** 10.1186/s40104-016-0096-6

**Published:** 2016-07-11

**Authors:** Yan Li, Di Lian, Shoulong Deng, Xiaosheng Zhang, Jinlong Zhang, Wenting Li, Hai Bai, Zhixian Wang, Hongping Wu, Juncai Fu, Hongbing Han, Jianzhong Feng, Guoshi Liu, Ling Lian, Zhengxing Lian

**Affiliations:** Key Laboratory of Animal Genetics and Breeding of the Ministry of Agriculture, Beijing Key Laboratory for Animal Genetic Improvement, College of Animal Science and Technology, China Agricultural University, Beijing, 100193 China; Department of Public Health, Benedictine University, Lisle, IL 60532 USA; State Key Laboratory of Reproductive Biology, Institute of Zoology, Chinese Academy of Sciences, Beijing, 100101 China; Tianjin Institute of Animal Sciences, Tianjin, 300381 China

**Keywords:** Growth, Microinjection, Pronuclear embryos, Sheep, Superovulation

## Abstract

**Background:**

Brucella is a zoonotic Gram-negative pathogen that causes abortion and infertility in ruminants and humans. TLR4 is the receptor for LPS which can recognize Brucella and initiate antigen-presenting cell activities that affect both innate and adaptive immunity. Consequently, transgenic sheep over-expressing TLR4 are an suitable model to investigate the effects of TLR4 on preventing Brucellosis. In this study, we generated transgenic sheep overexpressing TLR4 and aimed to evaluate the effects of different seasons (breeding and non-breeding season) on superovulation and the imported exogenous gene on growth.

**Results:**

In total of 43 donor ewes and 166 recipient ewes in breeding season, 37 donor ewes and 144 recipient ewes in non-breeding season were selected for super-ovulation and injected embryo transfer to generate transgenic sheep. Our results indicated the no. of embryos recovered of donors and the rate of pronuclear embryos did not show any significant difference between breeding and non-breeding seasons (*P* > 0.05). The positive rate of exogenous TLR4 tested were 21.21 % and 22.58 % in breeding and non-breeding season by Southern blot. The expression level of TLR4 in the transgenic sheep was 1.5 times higher than in the non-transgenic group (*P* < 0.05). The lambs overexpressing TLR4 had similar growth performance with non-transgenic lambs, and the blood physiological parameters of transgenic and non-transgenic were both in the normal range and did not show any difference.

**Conclusions:**

Here we establish an efficient platform for the production of transgenic sheep by the microinjection of pronuclear embryos during the whole year. The over-expression of TLR4 had no adverse effect on the growth of the sheep.

## Background

Transgenic animals are produced by introducing exogenous DNA into the genetic material of pre-implantation embryos to enhance their usefulness to humans [1]. Microinjection of exogenous genes is a common method to produce transgenic animals. The production of the first transgenic sheep was reported in 1985 via microinjection of exogenous gene into the pronucleus [2]. However, the site of integration is random, therefore the exogenous gene may be expressed poorly or inappropriately, which may result in some essential genes disrupted or oncogenes activated [3]. Recently, the good news is that a new technology called CRISPR/Cas9 would achieve the site-specific integration of the exogenous gene that will help resolve this defect [4–7]. The efficiency of microinjection has been a limitation to the development of transgenic sheep models. Only 5–8 % of the offspring carrying exogenous gene using usual pronuclear microinjection (The positive rate was calculated by the no. of positive animals/the no. of total born animals) [8–11]. Our previous study optimized the structure of exogenous gene, the injection dose and making use of high-quality in vivo pronuclear embryos so that achieving high positive rate (28.26 %) of pronuclear microinjection [12]. In this study, we optimized the superovulation procedure and hormone dosage which could obtain similar superovulation effect including the no. of embryos recovered and the production efficiency of pronuclear embryo in different seasons in order to achieve production of transgenic sheep by microinjection during the whole year.

Brucellosis is a world-wide zoonotic disease caused by *Brucella* spp., a Gram-negative facultative intracellular pathogen, leading to significant impact on public health and livestock industry. In domestic animals such as sheep and cattle, the outstanding manifestation of the pathology is abortion, stillbirth, orchitis, epididymitis, and infertility as a result of the colonization of placenta, fetal tissues and sexual organs [13]. In humans, patients infected with *Brucella* present undulant fever, weight loss, depression, hepatomegaly, splenomegaly and other complications such as arthritis, spondylitis, orchitis, and endocarditis [14]. Humans are usually infected following contact with infected animals, ingestion of contaminated milk, milk products, meat or the inhalation of infected aerosolized particles. A relatively low dose of bacteria (10–50 bacteria) may make human become infection [15], and more than 500,000 new reported cases in humans each year [16]. Currently there are no efficacious human vaccines available [17].

As in other gram-bacteria, the lipopolysaccharide (LPS) of *Brucella* is an important component of the outer membrane [18], and TLR4 is essential for the initial responses of macrophages against LPS. It has been reported that *Brucella* signals through TLR2 and TLR4 [19]. Our previous study produced TLR4 transgenic sheep and we found macrophage cells from TLR4 transgenic sheep showed a high resistance to LPS [12]. Subsequent study indicated that TLR4 transgenic sheep had lower load of *Brucella* (Not yet published). In this study, we mainly investigated the effect of seasons on superovulation and in order to effectively produce embryos during the whole year for the production of transgenic sheep. Additionally, the differences of TLR4 transgenic sheep and non-transgenic sheep on body weight, body size and blood physiological parameters were analyzed. According to the Food and Agriculture Organization (FAO), transgenic biosafety evaluation means evaluating the safety of gene manipulation, effect of imported exogenous gene on the safety of recipient animal, the health of transgenic animals, and the safety of product from transgenic animal. This preliminary study will lay a foundation for biosafety evaluation of transgenic sheep.

## Methods

### Animals

This trial was performed at an experimental station of China Agricultural University. It was located in Beijing, China, at 40.13° north latitude and 116.65° east longitude. The whole procedure was carried out in strict accordance with the protocol approved by the Animal Welfare Committee of China Agricultural University (Permit No.: XK662). A total of 80 and 280 Suffolk sheep at one to three years old were used as donors and recipients, respectively, during the natural breeding season in October (24 donors, 90 recipients) and November (19 donors, 76 recipients) and the non-breeding season in May (22 donors, 79 recipients) and June(15 donors, 65 recipients). It is important to note that 30 donors turned to be recipients after superovulation therefore there were 310 recipients in total. The sheep were provided by Beijing Aoxin Animal Husbandry Company Co., Ltd (Beijing, China).

### Superovulation

Eighty (43 sheep in breeding season and 37 sheep in nonbreeding season) sheep were treated with CIDR + FSH. Oestrus was synchronised by 12-day insertion of CIDR devices (Eazi-Breed CIDR, InterAg, Hanilton, New Zealand). Each sheep was superovulated under the treatment of 230 IU FSH (Ningbo Sansheng Pharmaceutical Co., Ltd., Ningbo, China) starting on the ninth day. The FSH consisted of eight decreasing doses, two times per day. The CIDR were removed at the time of the seventh FSH treatment. During 24 h and 48 h after the CIDR removal, the donors were tested using vasectomized rams to confirm their oestrus, and each ewe was injected 130 IU LH at 2 h after testing (Ningbo Sansheng Pharmaceutical Co., Ltd., Ningbo, China). Laparoscopic uterine horn insemination was carried out with fresh diluted semen from two Suffolk rams at 54 h after CIDR withdrawal. The oocytes/embryos were flushed out from the oviduct by laparotomy and collected in petri dishes at 16 h after the insemination. The number of oocytes/embryos was examined for each donor under a stereomicroscope and briefly centrifuged (10,000 × g, 10 min) to visualize pronuclei to confirm fertilization.

### Microinjection and embryo transfer

The linearized TLR4 fragment vector solution consisted of the DNA and ddH_2_O (5pL, referring to EPPendorf femtoject) was injected into the transferable embryos that zona pellucida was clear, cytoplasm was uniformed and pronucleus was visible, under a final concentration of 10 ng/μL, which approximately contain 2.39 × 10^-8^ pmol of TLR4. Following microinjection, embryos were placed into holding medium and transferred to recipients within one hour after begining the laparotomy operation. Out of 280 recipients, 30 ewes were donors reused as recipients after superovulation above. The recipients were synchronized by 12-day insertion of CIDR devices which were removed 10 h earlier than that in donors, and each recipient was injected 280 IU PMSG (Ningbo Sansheng Pharmaceutical Co., Ltd., Ningbo, China). At 36 h after the CIDR withdrawal, oestrus was detected by using vasectomized rams. Two to four embryos were transplanted into ordinary recipient and the donors used also as recipient oviduct within one hour after begining the laparotomy operation. Pregnancy was diagnosed by transabdominal ultrasound scanning on the 60th day after embryo transfer.

### Identification of transgenic sheep and gene quantification

Transgenic sheep were identified by the Southern blot, using genomic DNA extracted from the ear tissue. Purified genomic DNA (20 μg) was digested with Hind III (NEB, Beverly, MA, USA) to obtain a fragment with 2771 bp in size which was transferred to Nylon membrane (Boehringer Mannheim, Germany). PCR amplification was used to generate a specific digoxigenin-labeled probe (Roche Diagnostics, Mannheim, Germany). The size of the TLR4 probe used for southern hybridization was 671 bp, and the primers for TLR4 were as follows: forward 5′-ACTGGTAAAGAACTTGGAGGAGG-3′ and reverse 5′-CCTTCACGACATTCAACAGACC-3′.

The expression of TLR4 was determined by real-time PCR. Briefly, the blood of five male transgenic positive sheep and ten paternal half-sib male transgenic negative sheep at 6 month old were collected from the jugular veins, and monocytes were isolated from blood by a lymphocyte separation medium (TBD, Tianjin, China). RNA was extracted from monocytes using the RNeasy Plus Mini Kit (Qiagen), and then reverse-transcribed into cDNA using the RevertAid First Strand cDNA Synthesis Kit (Thermo). β-actin was used as the representative house-keeping gene for normalisation. The primers of TLR4 for qPCR were as follows: forward 5′-ATTTTACACCATATTGCCGTCT-3′ and reverse 5′-CCTTGCATTCCTTTGGCGAGA -3′, and the primers for β-actin were forward 5′-AGATGTGGATCAGCAAGCAG-3′ and reverse 5′-CCAATCTCATCTGCTTTTCTG -3′. The qPCR reactions were run on the Mx3000P instrument (Agilent Technologies, Santa Clara, CA, USA) and amplification data were analyzed using the Mx3000P software. The relative expression was determined using the comparative 2^−ΔΔCT^ method.

### Growth and blood parameters analysis

The body weight, body length, height and chest girth of five male transgenic positive sheep and ten paternal half-sib male transgenic negative sheep were measured at the age of 0, 1, 2 months, these sheep were all born singly. In addition, some blood physiological parameters of the five male transgenic sheep and ten male transgenic negative sheep at 6 month old were measured, including red blood cell (RBC), hemoglobin (HGB), hematocrit (HCT), mean corpuscular volume (MCV), mean corpuscular hemoglobin (MCH), mean corpuscular hemoglobin concentration (MCHC), platelet (PLT), white blood cell (WBC), segmented neutrophil percentage (SEG%), band neutrophil percentage (BAND%), monocyte percentage (MON%), lymphocyte percentage (LYM%), eosinophil percentage (EOS%) and basophil percentage (BAS%). The blood physiological parameters were recorded in RA-1000 auto-analyzer (Technicon, Tarrytown, NY, USA).

### Statistical analysis

In this study, the sample size for breeding season and non-breeding season is 43 and 37, respectively. Independent samples t-tests (SAS Institute, US) were used to compare all results except the no. of left and right oviduct embryos recovered which were assessed by a one-way analysis of variance (ANOVA) and the comparison of the lambing rate between reuse of donors as recipients and ordinary recipient was performed by the *χ*^2^-test. There were four observations (Breeding season: 2, non-breeding season: 2) of pregnancy rate and lambing rate for every classification of CL no. (1, 2, 3) and transferred embryos (2, 3, 4), respectively. The pregnancy rate and the lambing rate also analyzed by the ANOVA. The rate of multiplets was analyzed as above. Results were expressed as mean ± SD and differences were considered significant at *P* < 0.05.

## Results

### The effects of different seasons on superovulation of donors

**Fig. 1:**
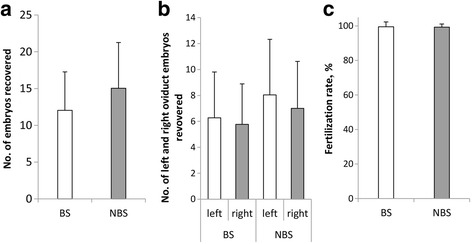
The effects of different seasons on superovulation of donors. **a** No. of embryos recovered. **b** No. of left and right oviduct embryos recovered. **c** Fertilization rate. BS = Breeding Season, *N* = 43. NBS = Non-breeding Season, *N* = 37

In the breeding season, 515 zygotes and 3 unfertilized ova were flushed out from 43 donor ewes. In the non-breeding season, 552 zygotes and 5 unfertilized ova were flushed out from 37 donor ewes. There was no significant difference of no. of embryos recovered (12.05 ± 5.22 versus 15.05 ± 6.22, *P* > 0.05, Fig. 1a) between sheep from breeding season and non-breeding season. Subsequently, we further compared the difference of ovulation in left and right ovary, and the results showed that there were no significant differences in both left and right oviduct between breeding and non-breeding season (Fig. 1b), as the fertilization rate (99.48 ± 2.84 % versus 99.29 ± 1.83 %, *P* < 0.05, Fig. 1c).

### The production efficiency of in vivo pronuclear embryos in different seasons

**Fig. 2:**
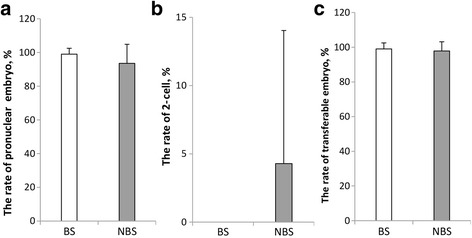
The production efficiency of in vivo pronuclear embryos in different seasons. **a** The rate of pronuclear embryo. **b** The rate of 2-cell. **c** The rate of transferable embryo. BS = Breeding Season, *N* = 43. NBS = Non-breeding Season, *N* = 37. Superscript letter (*) represents statistically significant difference (*P* < 0.05)

In brief, 512 transferable embryos (512 pronuclear embryos, 0 2-cells) and 3 untransferable embryos (In poor quality such as degeneration) were obtained in the breeding season. In addition, 542 transferable embryos (519 pronuclear embryos, 23 2-cells) and 10 untransferable embryos were obtained in the nonbreeding season. The 2-cells embryos were flushed out from the oviduct in vivo during the superovulation operation. They were resulted from cleavage of pronuclear embryo in vitro. For each sheep, the ratio of pronuclear embryos was calculated as the formula: (The no. of pronuclear embryos)/(Total no. of embryos flushed out of oviduct). The average and standard deviation were calculated. The ratio of 2-cells embryos was calculated as above. The transferable embryos included pronuclear and 2-cells embryos. The ratio of transferable embryos was calculated as above too. As shown in Fig. 2, higher rate of pronuclear embryos was observed in the breeding season but there was no significant difference between them (99.03 ± 3.48 % versus 93.56 ± 11.28 %, *P* > 0.05, Fig. 2a). The rate of 2-cells embryo was lower in breeding season than that in the nonbreeding season but they showed no significant difference (0.00 ± 0.00 % versus 4.29 ± 9.74 %, *P* > 0.05, Fig. 2b). Furthermore, there were no significant difference in the rate of transferable embryos between different seasons (99.03 ± 3.48 % versus 97.85 ± 5.32 %, *P* > 0.05, Fig. 2c).

### Pregnancy and lambing

**Fig. 3:**
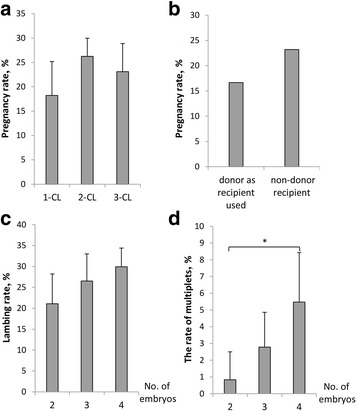
Pregnancy and lambing. **a** The pregnancy rate of different no. of corpus luteum of recipients (For each no. of CL, *N* = 4). **b** The pregnancy rate of reuse of donors as recipients and ordinary recipient. **c** The lambing rate of different no. of embryos transfered (For each no. of embryos, *N* = 4). **d** The rate of multiplets of different no. of embryos transfered (For each no. of embryos, *N* = 4). CL = Corpus Luteum. Superscript letter (*) represents statistically significant difference (*P* < 0.05)

In total, 13 of 78 recipients with one corpus luteum, 25 of 94 recipients with two corpus luteum and 20 of 85 recipients with three corpus luteum were pregnant, the other 53 recipients had more than three corpus luteum and we didn’t analyzed them on account of the small quantity. The pregnancy rate of recipients were 18.23 ± 6.95 %, 26.25 ± 3.71 % and 23.09 ± 5.79 %, respectively, and there were no significant differences among them (*P* > 0.05, Fig. 3a). In this study, after superovulation, 30 donors turned to be recipients and 5 sheep were pregnant. The pregnancy rate of the reuse of donors as recipients was 16.67 %, and the pregnancy rate of non-donor recipient was 23.21 % (65/280), there were no significant difference between them (Fig. 3b). In this experiment, 90 recipients were transplanted two embryos and 18 sheep were born including one double-lamb; 106 recipients were transplanted three embryos and 28 sheep were born including three double-lamb; 114 recipients were transplanted four embryos and 34 sheep were born including six double-lamb. The lambing rate were 21.08 ± 7.13 %, 26.53 ± 6.48 % and 29.94 ± 4.46 %, respectively, and there was no difference between the recipients transplanting different no. of embryos (*P* > 0.05, Fig. 3c). The rate of multiplets were 0.83 ± 1.67 %, 2.78 ± 2.08 % and 5.47 ± 2.96 %, respectively, and transplanting four embryos got higher rate than transplanting three embryos (*P* < 0.05, Fig. 3d).

### Identification of exogenous TLR4 gene and quantification

**Fig. 4:**
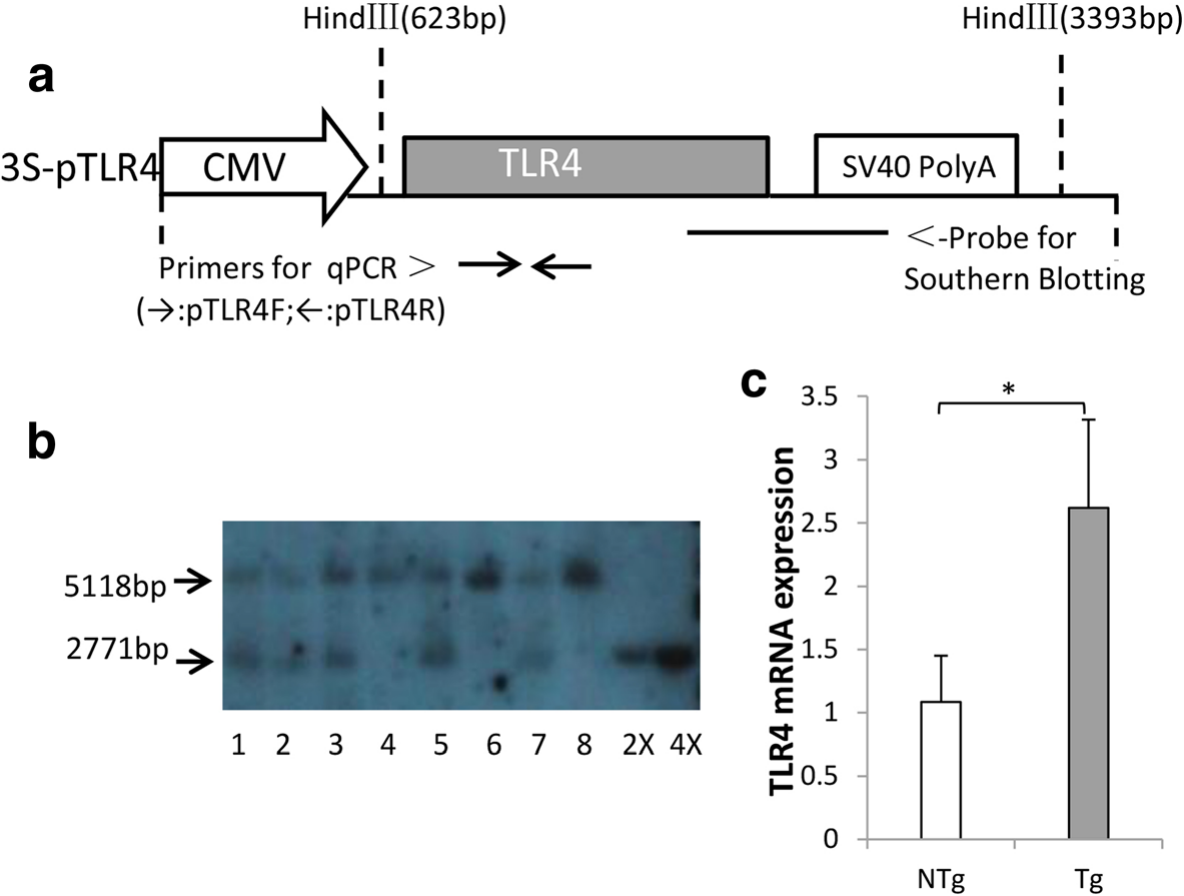
Identification of exogenous TLR4 gene and quantification. **a** The brief structure of 3S-pTLR4 vector, the sizes of the injected TLR4 fragment was 3172 bp. **b** Southern blot analysis. The 5118 band was the endogenous TLR4 fragment when the sheep genomic DNA was digest by Hind III. Numbered 1, 2, 3, 5 and 7 represent positive individuals. Numbered 4, 6 and 8 represent negative individuals. 2× and 4× are samples of transgenic vectors, here used as positive controls. **c** The mRNA expression of TLR4 was quantified using real-time PCR, each individual was repeated three times (*n* = 3). Tg = Transgenic sheep, *N* = 5. NTg = Non-transgenic sheep, *N* = 10. Superscript letter (*) represents statistically significant difference (*P* < 0.05)

As shown in Table 1, in total, 310 recipient sheep were transplanted and 80 lambs were born including six dead fetuses, the survival rate was 92.50 %. Subsequently, 64 sheep out of 74 live sheep (Ten sheep were used for other experiment, therefore they were removed from this study) were identified by Southern blot and 14 sheep were positive, the positive rate was 21.88 %, in which, seven transgenic sheep were produced at non-breeding season. Meanwhile, it’s worth noting that one positive sheep was resulted from the reuse of donors as recipients. By real-time PCR, the expression level of TLR4 in the transgenic sheep was 1.5 times higher than the non-transgenic group (*P* < 0. 05, Fig. 4c).

**Table 1:** Production of transgenic sheep over-expressing TLR4

Season	Number of recipients	Lambing rate, %	Survival rate, %	Positive rate, %
Breeding	166	27.71 (46/166)	93.48 (43/46)	21.21 (7/33)
Non-breeding	144	23.61 (34/144)	91.18 (31/34)	22.58 (7/31)
Total	310	25.80 (80/310)	92.50 (74/80)	21.88 (14/64)

### Growth and blood parameters analysis of transgenic and non-transgenic sheep

**Fig. 5:**
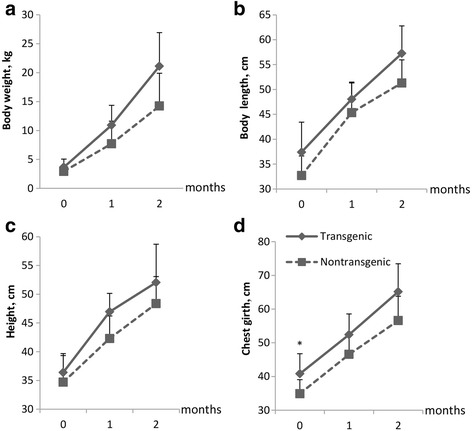
The comparison of growth traits between transgenic and non-transgenic sheep. **a** Body weight. **b** Body length. **c** Height. **d** Chest girth. The solid line represents transgenic lambs and the dotted line represents non-transgenic lambs. Tg = Transgenic sheep, *N* = 5. NTg = Non-transgenic sheep, *N* = 10

As shown in Fig. 5, the lambs over-expressing TLR4 showed similar growth performances to non-transgenic lambs. The blood physiological parameters between transgenic and non-transgenic sheep did not show any difference except HCT which was higher in the transgenic sheep than that in the non-transgenic sheep (Table 2, *P* < 0.05). However, both of them were in the normal range.

**Table 2:** The comparison of blood parameters between transgenic and non-transgenic sheep

Parameters	Transgenic	Non-transgenic	Normal range [42, 43]
Number of sheep	5	10	
RBC, 10^12^/L	10.67 ± 0.49	9.77 ± 0.85	8–15
HGB, g/L	108.67 ± 8.08	96.29 ± 8.46	80–160
HCT, %	36.73 ± 2.67*	30.07 ± 2.03	24–45
MCV, fL	32.53 ± 1.65	30.87 ± 2.02	23–48
MCH, pg	10.20 ± 0.56	9.87 ± 0.66	8–12
MCHC, g/L	312.67 ± 3.06	319.71 ± 8.44	290–350
PLT, 10^9^/L	348.00 ± 13.53	312.43 ± 139.79	175–500
WBC,10^9^/L	9.20 ± 0.61	8.87 ± 1.27	4–12
SEG, %	65.70 ± 4.35	46.73 ± 15.13	1–52
BAND, %	0.10 ± 0.01	0.09 ± 0.04	0–3
MON, %	2.43 ± 1.15	2.66 ± 1.73	1–5
LYM, %	27.13 ± 4.43	43.89 ± 14.90	40–75
EOS, %	3.40 ± 1.64	5.66 ± 3.13	1–8
BAS, %	1.33 ± 0.21	1.07 ± 0.51	0–3

Superscript letter (*) represents statistically significant difference (*P* < 0.05). Tg = Transgenic sheep, *N* = 5. NTg = Non-transgenic sheep, *N* = 10

## Discussion

The majority of sheep breeds are, like most ruminants, seasonal breeders and the breeding season is autumn such as Suffolk [20]. Some studies reported that superovulation is better in breeding season than that in non-breeding season [21, 22]. However, in this study, the no. of embryos recovered and the fertilization rate of sheep between breeding and non-breeding season showed no significant difference. Therefore better superovulation effect can be obtained both in the breeding and nonbreeding season using rational superovulation procedure and hormone dosage. In this study, uterine horn insemination was completed by endoscopic surgical technique, which provided relatively higher fertilization rate, compared to other insemination methods such as cervical insemination and vaginal insemination. Meanwhile, it can save the insemination time and budget as well as the reduction of the harm to the donors [23].

The production of high quality pronuclear embryos is key in producing transgenic sheep by microinjection. Simons et al. reported that all the transgenic sheep were developed from pronuclear embryos and the sheep developed from multicellular stage such as 2-cell and 4-cell were negative [24], which indicated the integration efficiency of exogenous gene was higher in pronuclear stage compared with multicellular stage. This was a potent evidence to show the importance of pronuclear embryo in producing transgenic sheep. In this study, high production efficiency of pronuclear embryos was obtained in different seasons with 93.56 % in non-breeding season and 99.03 % in breeding season. This was obviously higher than other researches such as reported by Guzik et al. [23] and Baldassarre et al. [25], also, this could provide sufficient in vivo pronuclear embryos for efficient production of transgenic sheep.

In this study, we investigated the effect of the no. of corpus luteum, reuse of donors as recipients and the no. of embryos transferred on the pregnancy and lambing rate in order to explore a best program of embryo transfer. The corpus luteum was a temporary endocrinal organ developed from ovulated follicle and it would secrete the progesterone. The main function of progesterone was to promote the embryo implantation and maintain the pregnancy [26]. We observed the no. of corpus luteum had no significant impact on pregnancy rate. Bari F et al. [27] and Evans A et al. [28] reported that quantity of CL. had no relationship with pregnancy rate which were consistent with our results, but the quality of corpus luteum directly affected the pregnancy rate. In this experiment, the donors used also as recipients after the superovulation surgery, they had several embryos transplanted into them immediately. Compared with the non-donor recipient, the pregnancy rate of reuse of donors as recipients did not significantly decrease. What’s more, one positive sheep was resulted from the reuse of donors as recipients which was a promising attempt. The reuse of donors as recipients could increase the utilization rate of sheep, save resources and increase the recipient number. Cseh et al. showed that increasing the no. of embryos transferred would increase the pregnancy rate [29]. We observed that the lambing rate was not significantly different for transplanting different no. of embryos. In general, the lambing rate of four embryos relatively higher than that of two and three embryos, meanwhile the rate of multiplets was also higher than others. Therefore, if the embryos were sufficient, transferring four embryos was better to maximize the transgenic offspring. However, when we took into account the cost and efficiency, transferring two embryos seemed much more efficient. In this case, by increasing the recipients no. also could get more offspring.

Microinjection is a commonly used method in the production of transgenic animals. Nonetheless, the production of transgenic animals by microinjection, in particular, is a very inefficient. This is an important limiting factor in production of transgenic animals. The percentage of gene-injected embryos that finally developed into transgenic sheep varied from 0.1 to 4 % which was calculated by the number of positive animals/the total number of injected embryos [30–33]. It was 1.46 % (or slightly higher because 16 sheep were not identified) in our study which is medium. In addition, another formula was used to calculate transgenic positive rate which is number of positive animals/the number of total born animals [8–11]. By this formula, 21.88 % of transgenic positive rate of exogenous TLR4 was obtained in this study which is higher than that in previous studies (5–8 %).

Toll-like receptors (TLRs) were a kind of type I transmembrane glycoproteins, playing a vital role in the innate immunity system by activating proinflammatory signaling pathways in response to microbial pathogens [34]. Toll receptors were first identified in studies of dorsal-ventral polarity formation of Drosophila embryo [35]. TLR4, one of the Toll-like receptor family, which recognized LPS and initiated a series of intracellular responses, and induced cytokine expression in a variety of cell types against Gram-negative bacteria [36]. TLR4 can activate nuclear factor-kappa B (NF-κB) by MyD88-dependent or MyD88-independent signal pathways [37]. Over-expression of TLR4 in transgenic animals could improve the disease resistance. Under LPS stimulation, over-expression TLR4 sheep rapidly activated the TLR4 signaling pathway and help the host launch an immune response against pathogen invasion and infection [12]. In addition, TLR4 plays a crucial role in resisting *Brucella*. TLR4 contributes to internalization and clearance of *Brucella* by macrophages [38–41]. In this study, we preliminary evaluated whether the imported exogenous gene TLR4 exerted an effect on biosafety of the transgenic sheep itself. The body weight, body size, and blood physiological parameters in both the transgenic sheep and non-transgenic sheep were similar. These preliminarily suggested that there was no adverse effect of TLR4 over-expression on the sheep, which laying a foundation for making deeper biosafety evaluation such as reproductive safety evaluation, 90-day feeding evaluation of transgenic sheep meat in rat and intestinal microflora evaluation.

## Conclusion

In summary, pronuclear embryos could be efficiently produced both in the breeding and non-breeding season which would achieve generating transgenic sheep by microinjection the whole year and the over-expression of TLR4 had no adverse effect to the growth of the sheep.
